# Perception and utilization of cervical cancer screening services among female nurses in University College Hospital, Ibadan, Nigeria

**Published:** 2012-04-15

**Authors:** Oyedunni Sola Arulogun, Opemipo Olubunmi Maxwell

**Affiliations:** 1 Department of Health Promotion and Education, Faculty of Public Health, College of Medicine, University of Ibadan, Ibadan, Nigeria; 2 Public Health Nursing Department, University College Hospital, Ibadan, Nigeria

**Keywords:** Female nurses, cervical cancer, cancer screening, utilization, perception, Nigeria

## Abstract

**Background:**

Cervical Cancer (CC) is the second most common cancer among women with early detection and prompt treatment as best management options. Female nurses have crucial roles to play in promoting the utilization of Cervical Cancer Screening Services (CCSS), yet little information exist regarding their perception and utilization of these services. The CCSS related knowledge, perception and utilization among female nurses at the University College Hospital, (UCH) Ibadan, Nigeria were therefore determined.

**Methods:**

A survey of 503 consenting nurses was done using a pretested self-administered questionnaire which included a 40-point knowledge scale and questions on perception of CC. Data were analyzed using descriptive statistics, Chi-square test, ANOVA and logistic regression.

**Results:**

Respondents’ mean age was 38.0 ± 8.6 years, mean year of experience was 12.5 ± 8.9 years and overall mean knowledge score was 22.8±4.1. Mean knowledge scores by cadre were Assistant Directors (26.7 ± 1.5), Chief Nursing Officers (23.4 ± 2.3) and Staff Nurses (21.7 ± 5.3) (p<0.05). Eighty-eight percent correctly perceived CC to be preventable and 82.0% believed that screening should be carried out as soon as sexual intercourse starts irrespective of age. Only 32.6% had ever used CCSS facility and main reasons for non-use included lack of time (50.8%), fear of result (13.9%) and not being sexually active (6.3%). Staff Nurses were four times less likely to utilize cervical screening services than the Assistant Directors of Nursing (OR 0.23, CI 0.117-0.442).

**Conclusion:**

Utilization of cervical cancer screening services among the female nurses was poor. Strategies that encourage utilization are hereby advocated.

## Background

Cervical cancer constitutes a major public health threat to women in many low and medium resourced countries in South and Central America, sub-Saharan Africa, South and Southeast Asia where it is still the leading type of cancer among women [1–3]. The high burden of cervical cancer in these countries is due both to a high prevalence of Human Papillomavirus (HPV) infection and the lack of effective cervical cancer screening programs. In cases where effective screening programs are available, poor knowledge and negative health seeking behaviour of the populace have led to poor utilization of such services [2].

It is estimated that over one million women worldwide currently have cervical cancer, most of whom have not been diagnosed, or have no access to treatment that could cure them or prolong their lives [4]. In the developing countries of the world, a large proportion of cervical cancers are diagnosed in advanced stages, with poor rates of survival [2, 5]. In addition, the incidence of cervical cancer begins to rise at age 20-29 years, reaches a peak around 55-64 years, and declines somewhat after 65 years. The age-standardized incidence rates during 1993-97 varied from 20-55 per 100,000 women in most of the regions in developing countries where incidence data were available [6].

Early detection and prompt treatment of cancer and pre-cancerous conditions provide the best possible protection against cancer. Well organized screening has been shown to be effective in the reduction of both mortality and morbidity from cancer of the cervix [7].

Cervical cancer ranks the second most frequent cancer among women in Nigeria after breast cancer and about 24.8% of women in the general population are estimated to harbour cervical HPV infection at a given time [8]. This is a source of concern in a country with a population of 36.6 million women aged 15 years and above who are at risk of developing cervical cancer [9]. However anecdotal evidence has shown that utilization of cervical cancer screening for the prevention of the disease is poor in Nigeria. This was buttressed by the summary of the activities at the colposcopy unit of antenatal clinic of the University College Hospital which showed that only 3,038 women were screened between 2005 and 2007 out of the large population that visited the Antenatal clinic and the hospital as a whole.

Health workers especially nurses are often times looked upon as "role models" in health related issues. Nurses play a major role in enlightening the public on the availability and need for cervical cancer screening services. They are informed individuals who are expected to have more information and knowledge about several health related issues and also act as role models in uptake of preventive services but studies have documented otherwise. In a study among nurses in Nnamdi Azikwe University Teaching Hospital, Nnewi, Nigeria on awareness of cervical cancer screening services, results showed that 87% were aware of the existence of cervical cancer screening services but only 5.7% had ever been screened for cervical cancer [10]. Their attitude to and practice relating to such issues might positively or negatively influence the decision made by the community members. It is therefore pertinent to appraise their perception and utilization of cervical cancer screening services. This study therefore set out to assess the perception and utilization of cervical cancer screening services among female nurses in University College Hospital, Ibadan, Nigeria.

## Methods

### Research Design

This descriptive and cross-sectional study aimed at assessing and documenting the perception and utilization of cervical cancer screening services among female nurses of the University College Hospital, Ibadan, Nigeria. It sought to understand the perception of this population about cervical cancer, its risk factors, severity and prevention.

### Study setting

University College Hospital (UCH) is located in Ibadan, southwest Nigeria and it is the first out of the 15 Federal University Teaching Hospitals in Nigeria and a referral centre. Service departments in UCH are grouped into clinical and non-clinical departments. The clinical department consisted of 16 departments (Anesthesia, Dental Surgery, Obstetrics & Gynaecology, Ophthalmology, Otorhinolaryngology, Radiology, General Surgery, Community Medicine, Family Medicine, Haematology, Internal Medicine, Microbiology, Paediatrics, Psychiatry, Chemical Pathology and Histopathology) and the non-clinical comprised 6 departments (Pharmacy, Physiotherapy, Nursing, Records, and Administrative/Finance Departments). The Nursing Department was used for this study.

### Sampling Procedure and sample size

Stratified, proportionate and simple random sampling techniques were adopted for the selection of the 503 nurses from a total of 1006 nurses from all cadres of nurses in the hospital.

### Instrument for data collection

A pretested self administered semi-structured questionnaire containing a 40-point knowledge scale and questions on nurses’ perception of CCSS was used for data collection.

### Data Collection Process

Each interview started with an introduction and overview of the research including the objectives of the study. The respondents were told not to write any name on the self-administered questionnaire. Respondents were encouraged to ask questions on what they do not understand in the questionnaire. Explanations were given to respondents as required to aid their understanding of unfamiliar terms. The questionnaires were retrieved back from each respondent immediately after completion and they were reviewed for completeness.

### Data Management, Analysis and Presentation

Administered copies of the questionnaire were edited and coded with the aid of a coding guide. Coded data were entered into a computer for analysis using the Statistical Package for Social Sciences (SPSS) version 15.0. In assessing knowledge level, every correct response was scored 1 and a wrong one was scored zero. These scores were pooled together to get a three-range level of knowledge. Those who scored between 0 - 19 points were categorized as having poor knowledge, those who scored 20 - 29 points were categorized to have an average level of knowledge and those with 30 - 40 points had good knowledge of cervical cancer and cervical screening services. Frequency counts were carried out on all the variables while categorical variables were analyzed using ANOVA and logistic regression.

### Ethical Consideration

The study followed the ethical principles guiding the use of human participants in research. Approval for the study was obtained from the University of Ibadan/ University College Hospital (UI/UCH) Health Research Ethics Committee. In addition, verbal informed consent was obtained from each respondent. All the respondents were informed that the survey was voluntary, and that they did not have to participate if they chose not to or could withdraw at any time. Respondents were assured that confidentiality of responses would be maintained during and after data collection. Only numbers were assigned to each copy of the questionnaire and no name was required on the questionnaire. The numbers were to facilitate data entry and analysis and no one can link the identity of the participants with the registration numbers.

## Results

### Socio-demographic characteristics of the respondents

A total of five hundred and three (503) nurses in the University College Hospital were surveyed. Majority 144(28.6%) of the respondents were staff nurses followed by nursing officers 136(27.0), principal nursing officers 85(16.9), chief nursing officers 70(14.0%), senior nursing officers 49(9.7%) while 19(3.8%) were assistant directors of nursing. Many 407 (80.9%) of the respondents were married, 85(16.9%) were single, 9(1.8%) were widowed and 2(0.4%) were divorced. The mean year of experience was12.5 ± 8.9 years.

### Respondents’ knowledge on cervical cancer

Majority 407 (80.9%) of the respondents knew that cervical cancer is the commonest cancer of the female reproductive tract and 54.5% correctly identified Papilloma Virus (HPV) as the primary cause of cervical cancer. Many of the respondents 188 (37.4%) said that cervical cancer mostly affects women from the age of 50 years and above, while 166 (33.0%) reported that cervical cancer is common in women between 40 and 49 years. Very few 35 (7.0%) said that cervical cancer is common in women below 30 years of age. Others are outlined in Table 1.

**Table 1:** Knowledge of Cervical Cancer among Nurses in University College Hospital

Cancer of the Cervix is the Commonest Cancer of the Female Reproductive Tract	Percentage
Yes	80.9
No	11.1
Uncertain	8.0
**Primary cause in development of cervical cancer**	
HPV	54.5
HIV	2.0
Unknown	41.8
Old Age	1.7
**Most affected Age group**	
Below 30 years	8.0
30 – 39 years	21.7
40 – 49 years	33.0
50 and Above	37.3

Majority of the respondents knew that sexual intercourse at early age (77.9%), heredity 365(72.6%) and the Human Papilloma Virus (70.4%) were risk factors for cervical cancer while 41.9% erroneously mentioned HIV a risk factor for cervical cancer. Other risk factors identified included abortion 246(48.9%), old age 256(50.9%), tobacco smoking 223(44.3%) and radiation 308(61.2%).

When probed about symptoms associated with cervical cancer, most of the respondents mentioned bleeding from the vagina (89.7%), painful intercourse (87.9%), purulent vaginal discharge (87.9%) and loss of weight (73.4%). On severity of the disease, 90.1% of the respondents opined that cervical cancer can kill. On whether it could be prevented, 88.0% were in the affirmative and 70.6% said it can be treated.

Knowledge of prevention revealed that 51.7% opined that regular pap smear can be used to prevent cervical cancer, 30.8% reported early diagnosis, 13.9% mentioned avoidance of multiple sexual partners and 11.9% early treatment. Other ways of prevention mentioned included avoidance of sexual intercourse (8.0%), health education (6.6%), and reduction in exposure to radiation (3.4%).

The overall analysis of knowledge of respondents showed that 84.9% had average knowledge score, 14.3% had poor knowledge and 0.8% had very good knowledge score. Respondents’ overall mean knowledge score was 22.8±4.1. These levels of knowledge were found not to influence the utilization of cervical screening services.

When asked what they would like to know about cervical screening techniques 399(79.3%) would like to know about screening procedures, 387(76.9%) about its efficacy in the detection of cervical cancer, 336 (66.8%) wanted to know the age limit for cervical cancer screening, 367(73.0%) about the side effects of the screening technique while 377(75.0%) would want to know what is next line of action after the screening.

### Awareness of Cervical Cancer Screening Service Centres

Four hundred and twenty-eight (85.1%) of the respondents were aware of at least one screening centre in the city of Ibadan. Majority, 465(92.4%) of the respondents were aware of the test called pap smear and 360 (77.4%) of these correctly stated what the test was used for the detection of cancer of the cervix and 455(90.5%) confirmed pap smear to be a diagnostic test. When asked at what age a woman should commence screening for cervical cancer, 81.7% mentioned that when a woman starts having sex and 62(12.3%) gave an age range of 15-39 years. When asked how often the screening should be done, 30.2% said twice a year and 38.0% of the respondents said once a year.

### Pattern of utilization of cervical cancer screening services

Table 2 shows the respondents’ pattern of utilization of cervical cancer screening (CCSS) services. Only 174 (34.6%) of the respondents had made use of cervical cancer screening services. Of these 174, 10.3% were Assistant Directors of Nursing, 21.8% were Chief Nursing Officers and 8.0% were staff nurses. Cadre of nurses was found to be significantly associated with utilization (p<0.05) (Table 3) Pattern of utilization showed that 80 (46.0%) had accessed CCSS only once, 48(27.6%) twice, 15(8.6%) thrice and 31(17.8%) four or more times with the University College Hospital being the mostly patronized (85.6%). Logistic regression analysis showed that staff nurses and nursing officers were four and almost five times less likely to utilize cervical cancer screening services than assistant directors of nursing (OR 0.233, CI 0.117-0442; OR 0.208, CL 0.092-0.0473) (Table 4).

**Table 2:** Patten of Utilization of cervical cancer Screening Service among Nurses in University College Hospital

Ever screened	Percentage
Yes	34.5
No	65.4
**Cadre of respondents ever screened**	
Assistant Director of Nursing	94.7
Chief Nursing Officer	54.3
Principal Nursing Officer	55.3
Senior Nursing Officer	32.7
Nursing Officer	30.1
Staff Nurse	9.7
**Number of times ever screened**	
Once	46.0
Twice	27.6
Thrice	8.6
4 or more times	17.8
**Reasons for non-screening**	
Lack of time	46.5
Fear of the result	12.8
Procedure is cumbersome	10.9
Cost consideration	8.2
Lack of awareness about where it can be done	8.8
Not sexually active	6.4
Lack of awareness of the test	6.4
**Likelihood for future screening**	
Yes	81.0
No	19.0

**Table 3:** Relationship between cadre of nurses and utilization of cervical cancer screening services

Cadre of Nurses	Utilization		X^2^	Df	P-value
	**Yes**	**No**			
Assistant Director of Nursing	94.7	5.3	104.4	5	0.000
Chief Nursing Officer	54.3	45.7			
Principal Nursing Officer	55.3	44.7			
Senior Nursing Officer	32.7	67.3			
Nursing Officer	30.1	69.6			
Staff Nurse	9.7	90.3			

**Table 4:** Logistic Regression showing the relationship between cadre of nurses and the utilization of screening services

Cadre	F	Df	Sig	Exp	95% CL ExpB	
					**Lower**	**Upper**
Assistant Director Nursing	19	5	0.000	RC*		
Chief Nursing Officer	70	4	0.000	0.006	0.001	0.050
Principal Nursing Officer	85	3	0.000	0.082	0.039	0.171
Senior Nursing Officer	49	2	0.025	0.076	0.037	0.154
Nursing Officer	136	1	0.000	0.208	0.92	0.473
Staff Nurse	144	1	0.00	0.233	0.117	0.442

Main reasons cited by the 329 who had never used cervical cancer screening services included lack of time 153(46.5%), fear of the result 42(12.8%), cumbersome procedure 36(10.9%), lack of awareness of where the test can be done 29(8.8%), cost consideration 27(8.2%), not sexually active 21(6.4%) and not knowing about the test 21(6.4%). Likelihood of going for screening was indicated among 409(81.0%) respondents. Significant others who reportedly can influence respondents’ decision to go for screening in a multiple response question were husbands (58.1%), doctors (49.5%) and colleagues (48.3%).

## Discussion

The level of knowledge found in this study was at variance with previous studies [11, 12] where majority of the study participants were not aware of cervical cancer. This is not unconnected with the profession of the respondents in this study where they are expected to be more knowledgeable than women in the community. The findings however affirms Udigwe's [10] assertions that in any community, trained nurses and midwives constitute a knowledgeable class with regards to medical information and intervention and that nurses are important health personnel that are supposed to educate women on the need for cervical screening.

The age range of women mostly affected by cervical cancer as reported by respondents in this study is in line with the findings of Guintoli and Bristow [13], where old age was identified as one of the risk factors associated with cervical cancer. Despite the level of knowledge on cervical cancer among the study respondents, gaps in knowledge still exist about other risk factors for cervical cancer. Majority of the respondents were of the opinion that only promiscuous women are at risk of cervical cancer. This is a misconception because not only promiscuous women are at risk of the disease. Women who are faithful but whose husbands visit sex workers are equally at risk of being infected with HPV as they might be infected by their husbands [14]. Women whose husbands have also been infected in the past are also at risk of being infected with the Human Papilloma Virus. This notion has to be corrected in intervention programs as it could lead to stigmatization and wrong labeling of those who are suffering from the disease as being promiscuous and could be a big barrier to women accessing screening services.

The finding that respondents’ knowledge on prevention and early detection of cervical cancer through Pap smear was low corroborates those of Awodele et al [15] who also documented a similar low level of knowledge among nurses studied in another teaching hospital in Nigeria. Poor utilization documented in this study also affirms the findings from the Nnewi study among nurses [10]. Reasons given for non-utilization such as fear of the results and not being candidate for cervical cancer have been documented in earlier studies in Uganda [16] and Owerri, Nigeria [17]. These misconceptions need to be addressed in an intervention program targeting this category of health workers. In addition the differentials that occurred among different cadre of nurses studied in the utilization of screening services is not unexpected. Younger nurses are not likely to use the service because they may perceive themselves as young, not susceptible and therefore not bothered about such issues. Anecdotal evidence has shown that the older a person becomes, the more concerned he or she is about his or her health and older women are therefore more likely to want to take preventive action.

The finding where most of the respondents mentioned husbands as significant person to influence screening behavior highlights the importance of male involvement in women's reproductive health issues, an emerging trend in reproductive health service utilization. The role of health practitioners was also reiterated by respondents’ indication of doctors and colleagues being significant others that could influence screening behavior. This finding cannot be over-emphasized as looking up to health professionals for health information and guidance is a norm in many societies. Friends were also chosen as people who could influence respondents screening behaviour which affirms the findings of Ezem [17] and Daramola [18] where friends documented as an informal but important source of health information in providing health information about cervical cancer.

One of the key implications of this study is the need for cervical cancer screening education programs to be carried out among health professionals at all levels especially among nurses. Despite the high level of awareness among the respondents, utilization remains low. Reproductive health education specialists have a significant role to play in reversing this trend among nurses as they constitute one of most authoritative sources of information about health matters for the general populace especially women. The continuing education program such as institution based health workshops and seminars provide an opportunity for doing this. Nurses need to be trained not only to provide comprehensive health education services routinely to their clients but to also motivate themselves to practice what they teach and lead by example.

## Conclusion

This study reveals that knowledge and utilization of cervical cancer screening services among the female nurses at the University College Hospital (UCH), Ibadan is low. Upgrading the knowledge base of nurses therefore becomes imperative as they play an important role in the prevention of cervical cancer in the community. To improve level of utilization, screening programs should work with husbands of women to promote early detection of cervical cancer among their wives and in this case nurses. Professional cadre and by extension social class is a variable to consider in improving utilization of screening services. The older nurses should be used encouraged to serve as cervical cancer screening motivators for their junior colleagues. This would ultimately dovetail to the community at large.

## References

[1] Parkin DM, Whelan SL, Ferlay J, Storm H (2005). Cancer Incidence in Five Continents, Volumes I to VIII IARC CancerBase No. 7, Lyon. http://www.iacr.com.fr/statist.htm.

[2] Sankaranarayanan R, Budukh AM, Rajkumar R (2001). Effective screening programmes for cervical cancer in low- and middle-income developing countries. Bull World Health Organ..

[3] Ferlay BF, Bray F, Pisani P, Parkin DM (2004). GLOBOCAN 2002: Cancer Incidence, Mortality and Prevalence Worldwide. IARC Cancer Base No. 5 Version 2.O. http:www-dep.iarc.fr.

[4] World Health Organization (2006). Comprehensive cervical cancer control: A guide to essential practice. http://whqlibdoc.who.int/publications/2006/9241547006_eng.pdf.

[5] Sankaranarayanan R, Thara S, Ngoma T, Naud P, Keita N, Finkel M (2010). Cervical Cancer Screening in the Developing World. Public Health in the 21st Century.

[6] Parkin DM, Whelan SL, Ferlay J, Storm H (2005). Cancer Incidence in Five Continents, vols. I-VIII. IARC Cancer Base no. 6. http://www-dep.iarc.fr.

[7] NHS Cervical Screening Programme, Cervical screening a pocket guide (2011). http://www.cancerscreening.nhs.uk/cervical/publications/cervicalpocket2009.pdf.

[8] Castellsagué S, de Sanjose T, Aguado KS, Louie L, Bruni J, Muñoz M, Diaz K, Irwin M, Gacic O, Beauvais G, Albero E, Ferrer S, Byrne FX (2007). HPV and cervical cancer in the 2007 report.

[9] Akinremi TO, Nazeer S, Totsch M (2005). Reduced alcohol use in the staining of Pap smears: a satisfactory low-cost protocol for cervical cancer screening. Acta Cytol.

[10] Udigwe GO (2006). Knowledge, attitude and practice of cervical cancer screening (pap smear) among female nurses in Nnewi, South Eastern Nigeria. Niger J Clin Pract..

[11] Phipps E, Cohen MH, Sorn R, Braitman LE (1999). A pilot study of cancer knowledge and screening behaviors of Vietnamese and Cambodian women. Health Care Women Int..

[12] Oladepo O, Ricketts OA, John-Akinola Y (2008). Knowledge and Utilization of Cervical Cancer Screening Services among Nigerian Students. International Quarterly of Community Health Education..

[13] Guintoli RL, Bristow RE, Cervical cancer, Gibbs RS (2008). Danforth's Obstetrics and Gynecology.

[14] Thomas JO, Herrero R, Omigbodun AA, Ojemakinde, Ajayi IO, Fawole A, Oladepo O, Smith JS, Arslan A, Munoz N, Snijders PJ, Meijer CJ, Franceshi S (2004). Prevalence of Papilloma Infection In Women In Ibadan, Nigeria: A Population Based Study. Br J Cancer.

[15] Awodele O, Adeyomoye AA, Awodele DF, Kwassh IO, Awodele IO, Dolapo DC (2011). A study on cervical cancer screening amongst nurses in Lagos University Teaching Hospital, Lagos, Nigeria. J Cancer Educ..

[16] Mutyaba T, Mmiro FA, Weiderpass E (2006). Knowledge, attitudes and practices on cervical cancer screening among the medical workers of Mulago Hospital, Uganda. BMC Medical Education..

[17] Ezem BU (2007). Awareness and Uptake of Cervical Cancer Screening in Owerri, South-Eastern Nigeria. Ann Afr Med.

[18] Daramola A (2001). A study of the awareness of screening procedure for carcinoma of the cervix (Pap smear) amongst health service users.

